# Hypothermia Prevents Retinal Damage Generated by Optic Nerve Trauma in the Rat

**DOI:** 10.1038/s41598-017-07294-6

**Published:** 2017-07-31

**Authors:** Manuel Rey-Funes, Ignacio M. Larrayoz, Daniela S. Contartese, Manuel Soliño, Anibal Sarotto, Martín Bustelo, Martín Bruno, Verónica B. Dorfman, César F. Loidl, Alfredo Martínez

**Affiliations:** 1 Laboratorio de Neuropatología Experimental, Instituto de Biología Celular y Neurociencia “Prof. E. De Robertis” (IBCN), Facultad de Medicina, Universidad de Buenos Aires, CONICET, Buenos Aires, Argentina; 2 Angiogenesis Study Group, Center for Biomedical Research of La Rioja (CIBIR), Logroño, Spain; 3 Laboratorio de Neurociencias, Facultad de Ciencias Médicas, Universidad Católica de Cuyo, San Juan, Argentina; 4 Centro de Estudios Biomédicos, Biotecnológicos, Ambientales y Diagnóstico (CEBBAD), Universidad Maimónides, Buenos Aires, Argentina

## Abstract

Ocular and periocular traumatisms may result in loss of vision. Hypothermia provides a beneficial intervention for brain and heart conditions and, here, we study whether hypothermia can prevent retinal damage caused by traumatic neuropathy. Intraorbital optic nerve crush (IONC) or sham manipulation was applied to male rats. Some animals were subjected to hypothermia (8 °C) for 3 h following surgery. Thirty days later, animals were subjected to electroretinography and behavioral tests. IONC treatment resulted in amplitude reduction of the b-wave and oscillatory potentials of the electroretinogram, whereas the hypothermic treatment significantly (p < 0.05) reversed this process. Using a descending method of limits in a two-choice visual task apparatus, we demonstrated that hypothermia significantly (p < 0.001) preserved visual acuity. Furthermore, IONC-treated rats had a lower (p < 0.0001) number of retinal ganglion cells and a higher (p < 0.0001) number of TUNEL-positive cells than sham-operated controls. These numbers were significantly (p < 0.0001) corrected by hypothermic treatment. There was a significant (p < 0.001) increase of RNA-binding motif protein 3 (RBM3) and of BCL2 (p < 0.01) mRNA expression in the eyes exposed to hypothermia. In conclusion, hypothermia constitutes an efficacious treatment for traumatic vision-impairing conditions, and the cold-shock protein pathway may be involved in mediating the beneficial effects shown in the retina.

## Introduction

Ocular globe and optic nerve trauma may occur in different daily scenarios including falls, motor vehicle accidents^1^, child abuse^2^, sport-related injuries^3^, as well as blasts in military and non-military combat situations^4^. Traumatic optic neuropathy (TON) may result in axon degeneration and retinal ganglion cell (RGC) death leading to profound visual loss.

Among the military population, blasts are the leading cause of disability resulting in traumatic brain and retinal injuries^5^. Blast-related injuries are due to the intense over-pressurization wave which applies intense forces to the tissues that result in their rapid deformation and disruption, as well as mechanical strikes by objects that are set in motion by the blast^5^. Visual impairment is a common symptom of blast-injured military veterans^6^. Specifying the effect of the blast wave on any part of the visual system is complicated by the complex nature of the injury. A part of the visual system could be directly injured or could degenerate secondary to distant injury via anterograde^7^ or retrograde^8^ mechanisms.

No effective treatments are currently available for these patients, although some experimental strategies, such as the upregulation of mTOR signaling^9^, are under study. Hypothermia, or exposure to cold temperatures, is being used clinically as a therapeutical intervention to reduce the symptoms of several pathologies, including stroke^10–13^, coronary artery bypass surgery^14^, neurodegeneration after cardiopulmonary resuscitation^15^, or neonatal asphyxia^16^, among others. Although therapeutic hypothermia is well accepted for the clinical treatment of cardiological or central nervous system complications^17^, at the moment this easy intervention is not used in humans for diseases of the eye. Nevertheless, a large number of animal studies has shown that hypothermia may be very useful in protecting the retina, especially in the context of ischemia^18–27^. Therefore, we propose that hypothermia may help in preventing the retinal damage associated to TON.

From all possible animal models of TON^28, 29^, we have chosen the intraorbital optic nerve crush (IONC) paradigm since we believe it better recapitulates traumatic retrograde injuries to the retina. We have also studied the expression of 2 cold-shock proteins (CSP), which increases after exposure to cold temperatures: RNA-binding motif protein 3 (RBM3) and cold inducible RNA-binding protein (CIRP or CIRBP). These proteins, which belong to the heterogeneous nuclear ribonucleoprotein family, bind to cellular RNAs and regulate their half-life and thus their expression potential and their final functions^30–34^. The expression of these proteins in the rat retina has been recently characterized^35^ and they may be part of the mechanism by which hypothermia prevents tissue damage.

Here we demonstrate that reducing eye temperature following IONC results in a significant prevention of vision loss, as measured by electroretinography, visual function, and morphological tests. In addition, expression of CSPs and anti-apoptotic signals were upregulated in the animals receiving hypothermic treatment.

## Results

**Figure 1:**
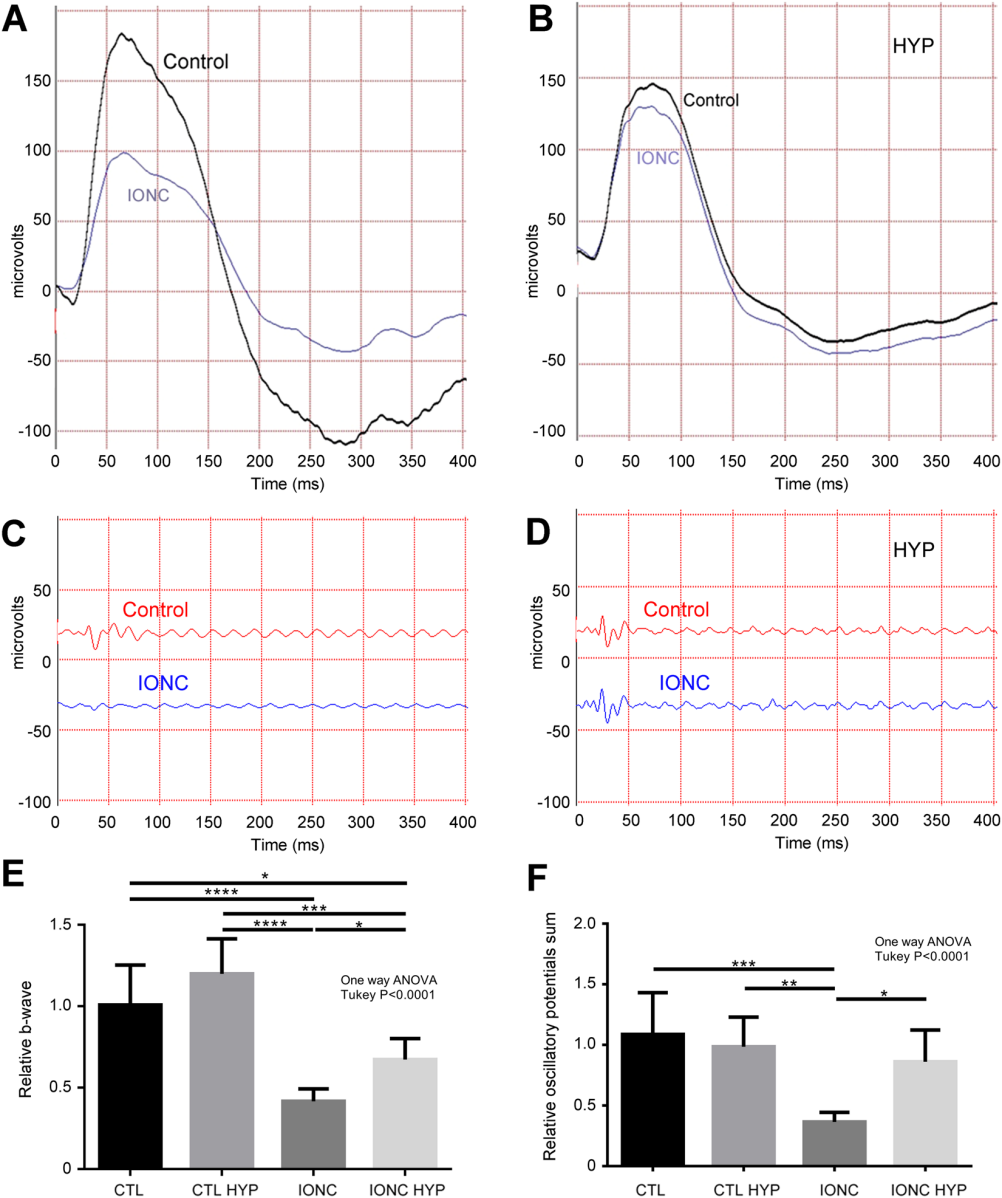
Representative electroretinograms showing the a- and b-waves (**A**,**B**) and the oscillatory potentials (**C**,**D**) of the right (sham-operated, control) and the left (IONC) eye of experimental rats kept at room temperature (**A**,**C**) or subjected to therapeutic hypothermia (**B**,**D**). Quantification of the relative (left eye/right eye) b-waves (**E**) and oscillatory potentials (**F**) are represented as histograms for the 4 experimental groups: sham-operated rats (CTL), sham rats treated with hypothermia (CTL HYP), rats subjected to IONC (IONC), and animals subjected to IONC and treated with hypothermia (IONC HYP). Bars represent the mean ± SD of all samples (n = 10 animals per group). Asterisks represent statistically significant differences. *p < 0.05; **p < 0.01; ***p < 0.001; ****p < 0.0001.

Experimental animals were subjected to IONC or sham-operated and then exposed to hypothermia (8 °C for 3 h) or left at normal room temperature (24 °C). After 3 h in hypothermia, animals had a retro-orbital temperature of 34.89 ± 0.16 °C whereas animals kept at regular room temperature had 36.53 ± 0.18 °C (p < 0.0001). Thirty days later, electric retinal currents were recorded in all animals by ERG. In rats kept in normothermia, the eye subjected to IONC presented a b-wave with lower amplitude than the sham-operated eye (Fig. 1A). In contrast, animals that were treated with hypothermia following the traumatic event had a b-wave more similar to the sham-operated eye (Fig. 1B). Quantification of this phenomenon shows a clear defect in IONC-treated eyes (p < 0.0001) that was significantly (p < 0.05) prevented by cold treatment (Fig. 1E). Something similar was observed when the OPs were analyzed (Fig. 1C,D). IONC-operated eyes presented a more regular OP pattern than their sham counterparts (Fig. 1C) but hypothermia treatment allowed the maintenance of a jagged pattern in IONC-operated eyes (Fig. 1D). This was also confirmed by quantification of the OPs that demonstrate a partial prevention of the IONC-induced defect (p < 0.05) (Fig. 1F).

**Figure 2:**
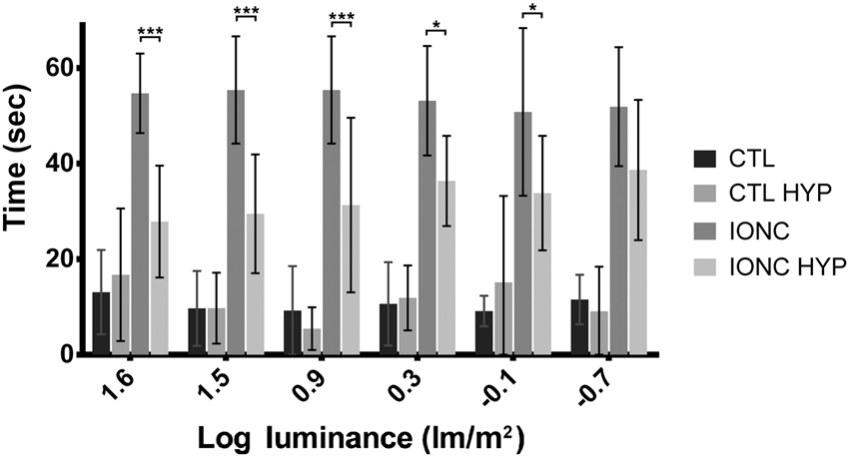
Time latencies needed for experimental rats to find the escape platform in the two-choice visual discrimination apparatus. The 4 experimental groups were sham operated rats (CTL), sham operated rats treated with hypothermia (CTL HYP), IONC operated rats (in both eyes), and IONC operated animals (in both eyes) treated with hypothermia (IONC HYP). Bars represent the mean ± SD of all samples (n = 10 animals per group). Asterisks represent statistically significant differences. *p < 0.05; ***p < 0.001; comparing IONC versus IONC HYP.

Visual function was assessed using a descending method of limits in a two-choice visual task apparatus designed to determine changes in the dark-adapted luminance sensitivity threshold. The task consisted in finding an escape platform submerged 2 cm in a circular tank containing water in the shortest possible period of time. Sham operated (control) animals were able to find the escape platform in about 10 sec and hypothermia applied to these animals did not have any significant influence in their visual discrimination capabilities (Fig. 2). Preliminary studies showed that animals with a single damaged eye relied heavily on the healthy eye and their maze performance was undistinguishable from control animals (results not shown). Thus we studied animals whose both eyes were IONC operated. Rats subjected to IONC in both eyes needed longer time (55 sec in average) to find the platform. In contrast, animals subjected to IONC (in both eyes) and treated with therapeutic hypothermia had a significantly shorter latency related to IONC (p < 0.001) under a wide range of illumination conditions (1.6 to −0.1 lm/m^2^) (Fig. 2).

**Figure 3:**
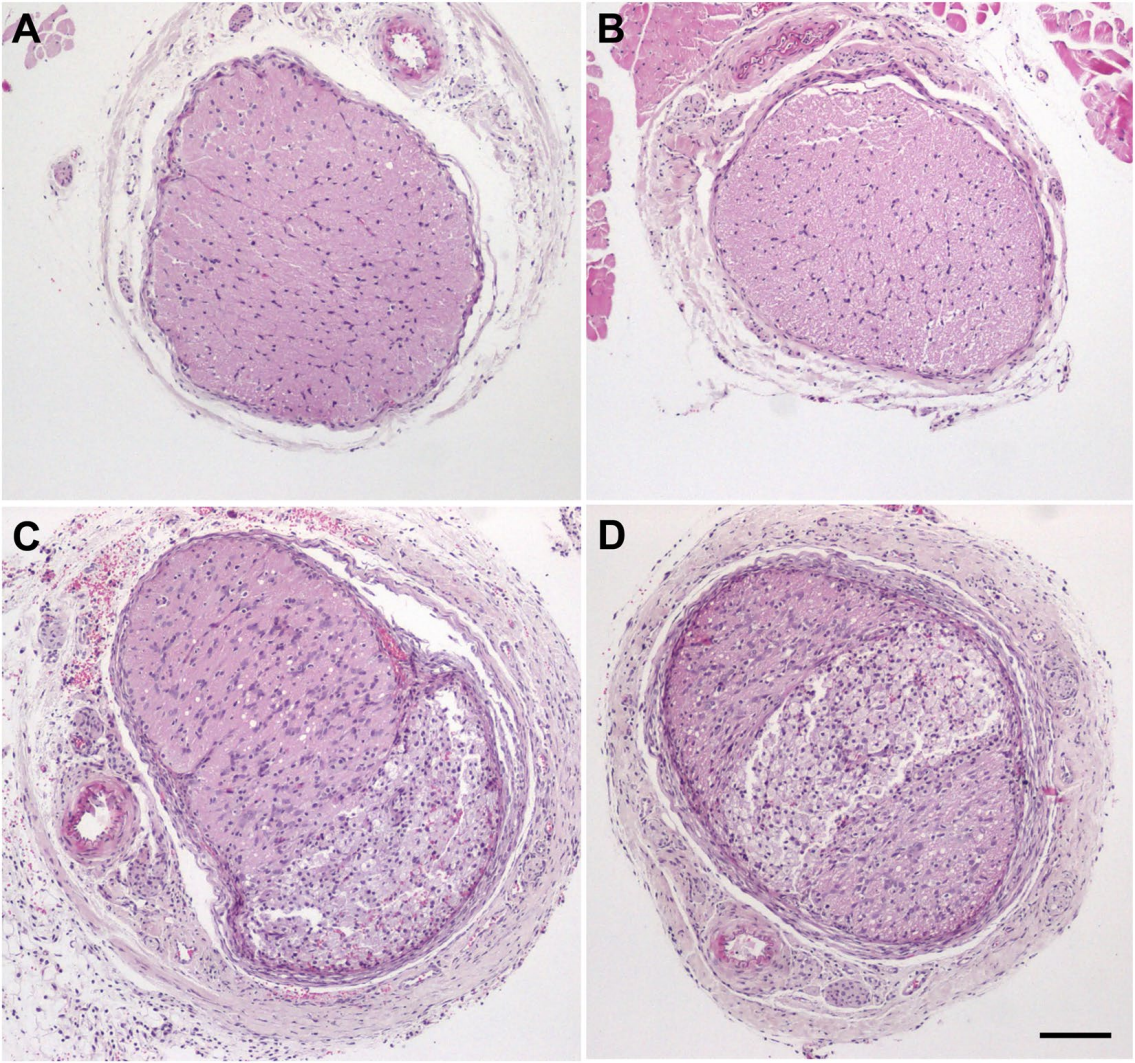
Representative histological images of the optic nerves of animals of the 4 experimental groups stained with hematoxylin & eosin: sham-operated (**A**), sham-operated treated with hypothermia (**B**), IONC-operated (**C**), and IONC-operated treated with hypothermia (**D**). Bar = 400 µm.

**Figure 4:**
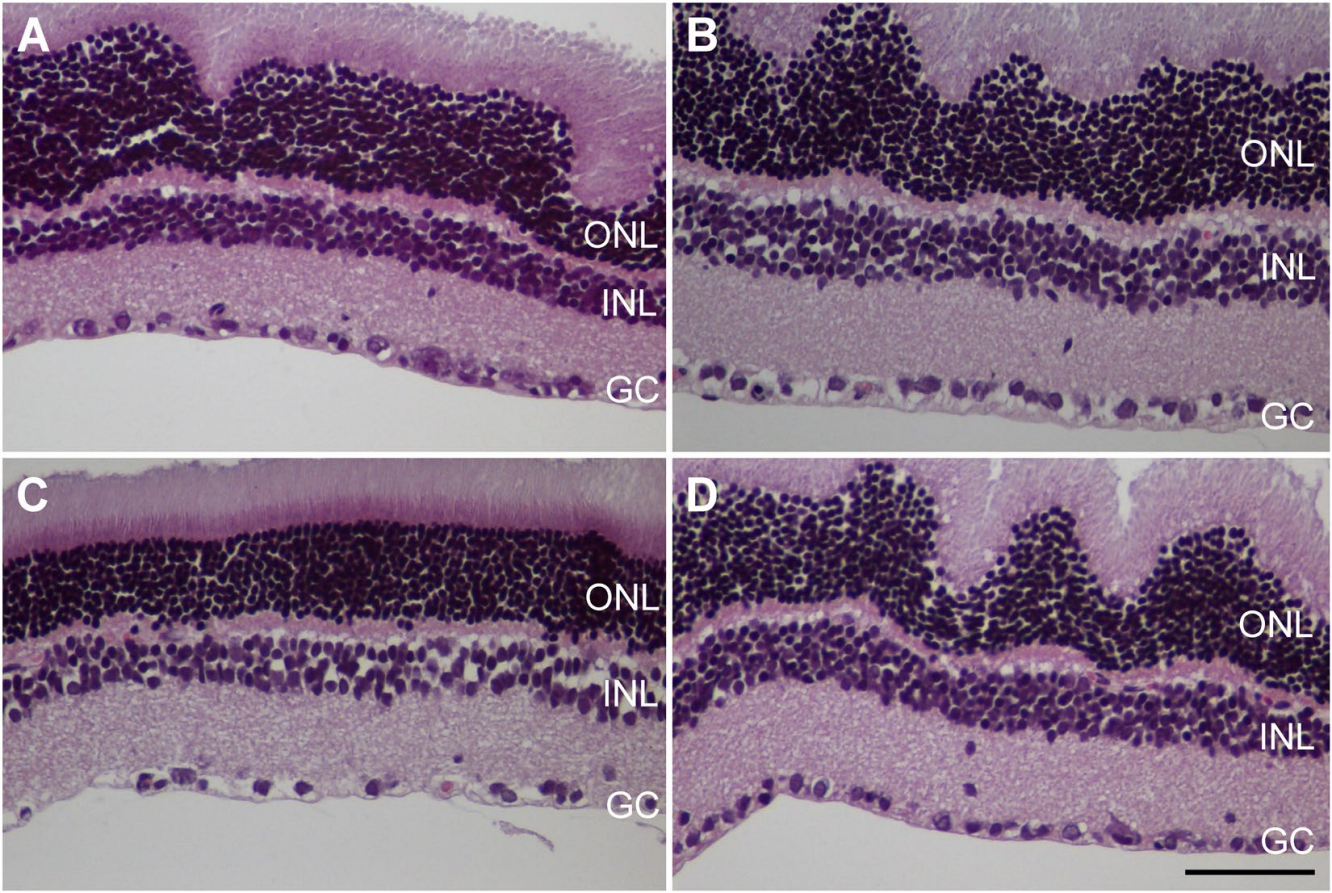
Representative histological images of the inner retina of animals of the 4 experimental groups: sham-operated (**A**), sham-operated treated with hypothermia (**B**), IONC-operated (**C**), and IONC-operated treated with hypothermia (**D**). Three layers of the retina are labeled in the pictures for reference: outer nuclear layer (ONL), inner nuclear layer (INL), and ganglion cell layer (GC). Bar = 100 µm.

**Figure 5:**
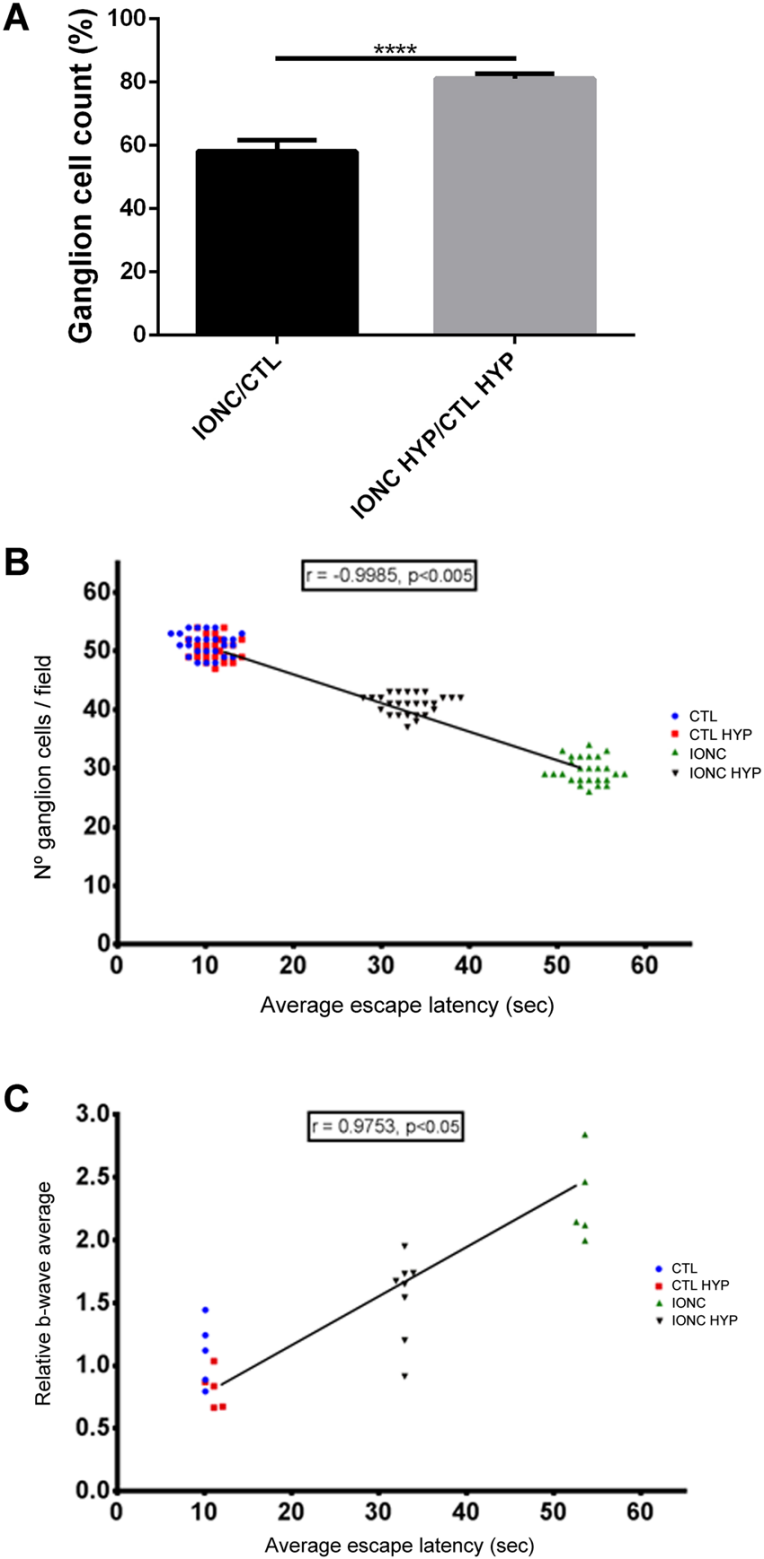
Variations in the number of RGCs and correlations between the average escape latency and the number of RGCs or the b-wave. (**A**) Number of ganglion cells per 25x objective microscopic field in the retina was determined and is represented as a ratio between the IONC-operated eye and its sham control. Hypothermia greatly reduced the destruction of RGCs. Bars represent the mean ± SD of all samples (n = 16 animals per group). Asterisks represent statistically significant differences. ****p < 0.0001. (**B**) Correlation study between the escape latency as measured in the modified water maze and the number of RGCs per microscopic filed. A highly significant correlation was detected with animals having more RGCs taking shorter times to find the escape platform. (**C**) Correlation study between the escape latency and the modification in the b-wave pattern observed by electroretinography. A significant positive correlation was detected with animals taking longer to find the escape platform having a more irregular electrophysiological pattern.

After the ERG and behavioral tests were completed by all animals, they were sacrificed and their retinas and optic nerves were collected for morphological analysis. As expected, optic nerves that had been subjected to IONC had clear morphological changes consistent with trauma, such as hypertrophy of the neural tubes (Fig. 3C) in contrast with the sham-operated eye (Fig. 3A). Cold exposure had no effect on the morphology of the optic nerves (Fig. 3B,D). Histological observation of the inner retina showed that IONC operated rats (Fig. 4C) had less RGCs than sham-operated animals kept under either normothermia (Fig. 4A) or hypothermia (Fig. 4B). Interestingly, IONC operated rats treated with therapeutic hypothermia (Fig. 4D) had more RGCs than their IONC counterparts (Fig. 4C). Quantification of the number of RGCs is presented in Fig. 5A as a ratio between the IONC eye and the sham control in the same animal. We also performed a correlation study between the number of RGCs and the escape latency measured in the two-choice visual discrimination apparatus (Fig. 5B) or between the escape latency and the relative change in b-wave observed with ERG (Fig. 5C). In both cases there was a significant correlation between those values indicating the expected connection among RGC death, electrophysiology of the retina, and visual perception by the animals.

**Figure 6:**
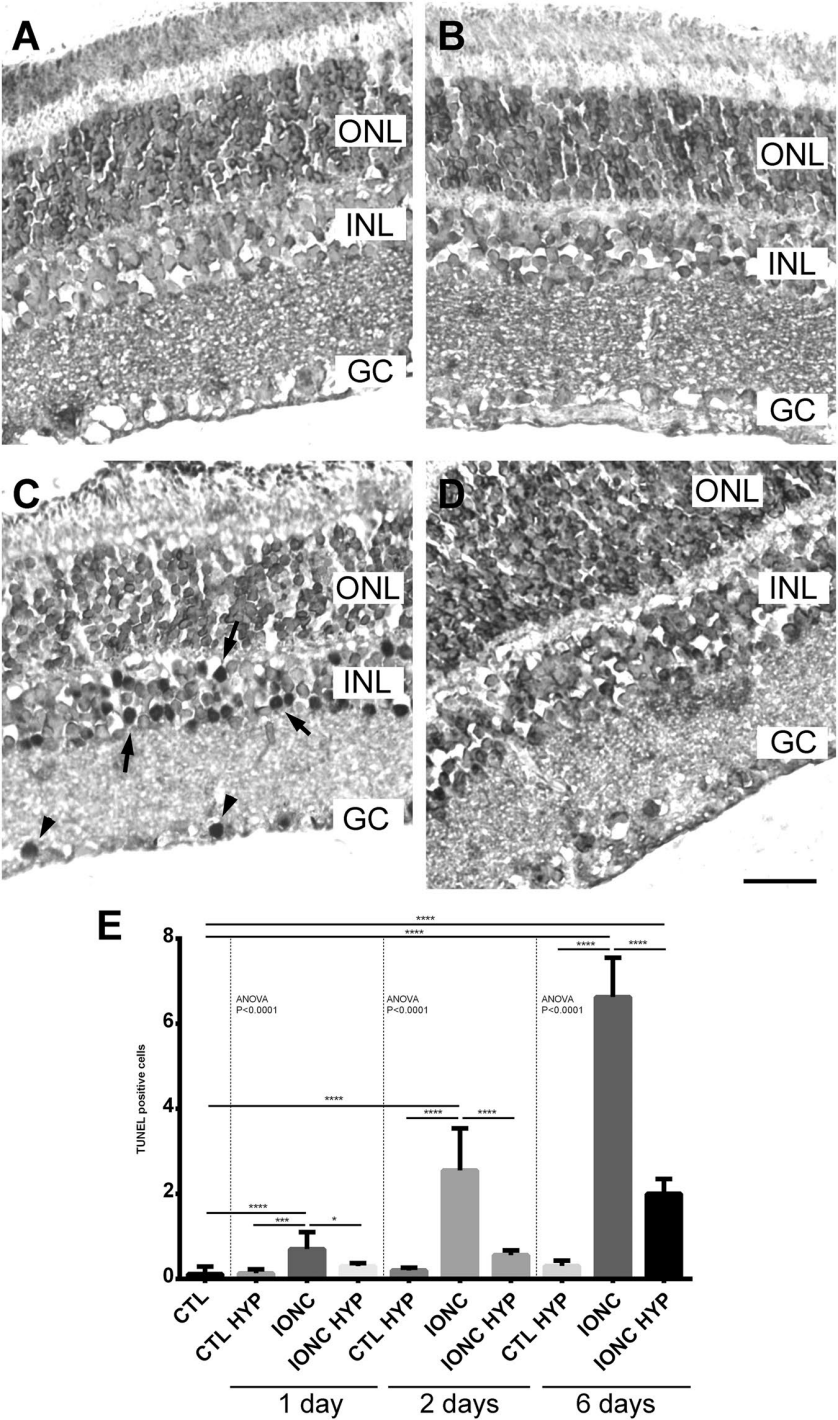
TUNEL positive cells in the 4 experimental groups 6 days post surgery. Representative images of retinas from sham-operated (**A**), sham-operated treated with hypothermia (**B**), IONC-operated (**C**), and IONC-operated rats treated with hypothermia (**D**). TUNEL positive cells were found mainly in the ganglion cell (GC) layer (arrowheads) and in the inner nuclear layer (INL, arrows). ONL: outer nuclear layer. Bar = 50 µm. Quantification of the results at 3 time points after surgery are shown as histograms (**E**). Bars represent the mean ± SD of all samples (n = 5 per group). Asterisks represent statistically significant differences. *p < 0.05; ***p < 0.001; ****p < 0.0001.

TUNEL analysis was performed in the retina of experimental animals at 1, 2, and 6 days after surgery. As expected, very few TUNEL positive cells were found in control animals whether exposed or not to hypothermia (Fig. 6A,B,E). A high number of TUNEL positive cells were found in the GCL and INL of the retinas of animals subjected to IONC (Fig. 6C,E), confirming the morphological data shown above. Here the application of hypothermia was also able to drastically reduce the number of TUNEL-positive cells when compared to the IONC only group, at all time points studied (Fig. 5D,E).

**Figure 7:**
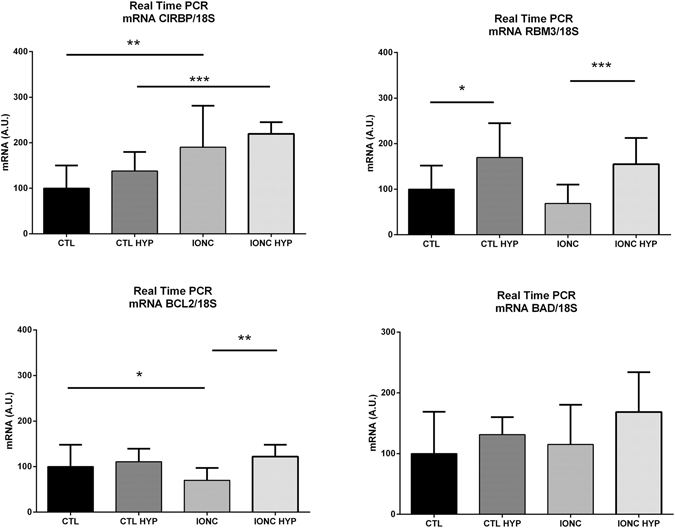
Relative gene expression for CIRP (**A**), RBM3 (**B**), BCL2 (**C**), and BAD (**D**) in the 4 experimental groups, as measured by qRT-PCR. All values were normalized using the house-keeping gene 18S. Bars represent the mean ± SD of all samples (n = 6 per group). Asterisks represent statistically significant differences, as indicated. *p < 0.05; **p < 0.01; ***p < 0.001.

To evaluate early genomic response to IONC and hypothermic treatment, gene expression analysis was performed by qRT-PCR in tissues collected 3h after completing hypothermic (or normothermic) treatment. CIRP expression was significantly elevated in IONC-treated eyes (p < 0.01). Cold treatment elicited a trend towards higher CIRP levels, although the differences did not reach statistical significance (Fig. 7A). On the other hand, RBM3 levels did not change due to IONC treatment but were significantly elevated by the cold treatment, specially in the eyes subjected to IONC (p < 0.001) (Fig. 7B). It has been shown that CSPs regulate gene expression for several proteins involved in the regulation of apoptosis^36, 37^, therefore we looked at the expression of representative genes. Expression of the antiapoptotic molecule BCL2 was significantly reduced (p < 0.05) in IONC-treated eyes when compared with sham controls. Interestingly, hypothermia significantly (p < 0.01) prevented the decrease of this gene following the traumatic event (Fig. 7C). On the other hand, expression of the proapoptotic gene BAD did not get significantly modified by IONC or hypothermia (Fig. 7D).

## Discussion

In this study we have shown that the visual loss subsequent to a traumatic event on the optic nerve can be partially prevented by application of hypothermia. This was demonstrated at various levels, including electroretinogram, visual behavior, and post-mortem morphological parameters. In addition, we have shown that CSPs are elevated in the eye following exposure to cold temperatures suggesting that they may be involved in mediating the beneficial effects elicited by hypothermia.

We have shown that IONC intervention results in a reduction of the b-wave and the OPs of the electroretinogram. This is in agreement with previous studies^38^. It is recognized that the a-wave is generated by the photoreceptors (outer retina), while the OPs and the b-wave represent electrical activities originating from the inner retina^39^. Different animal models of retinal damage influence electroretinograms in a model-specific fashion. For instance, ischemic retinopathy models are characterized by impaired inner retinal function and they present depressed b-waves in a way comparable to our model^39, 40^. Conversely, models of blunt ocular trauma or chemical intoxication induce photoreceptor apoptosis and a-wave disruption^41, 42^. In our model, depressed b-waves correlate with a significant reduction in the number of RGCs and with a longer latency in finding the hidden platform in behavioral testing, altogether indicating a poorer vision.

In the behavioral test, we observed that rats whose two eyes had been subjected to IONC took a long time to find the platform. In contrast, rats subjected to IONC and treated with hypothermia presented significantly shorter escape latencies under high illuminance conditions, confirming the beneficial effects of hypothermia.

Anatomically, we found a significant loss of RGCs in the eyes subjected to IONC. This is a common finding in this model due to retrograde damage^43, 44^. Application of hypothermia significantly prevented the destruction of RGCs, demonstrating its neuroprotective function in the retina, comparable to the effect elicited by well-established neurotrophic factors such as NT4, BDNF, or CNTF^45^. In addition, we showed a clear correlation between the number of RGCs in the retina and ERG and behavioral parameters, indicating that the 3 methods coincide in diagnosing the loss of vision in IONC-treated animals. The morphological data were confirmed by TUNEL analysis. Furthermore, this technique allowed us to identify the loss not only of RGCs but also of cells belonging to the INL. The reduction in the number of INL cells cannot be easily appreciated by regular staining techniques due to the high number of nuclei in this layer. The apoptosis of INL cells indicate that the retrograde damage affects not only RGCs, which are the cells whose axons have been damaged by the IONC procedure, but also bipolar, Müller, or amacrine cells further up the electrophysiological pathway. These data are also in agreement with the electroretinogram data that showed modifications in b-wave and OPs in the IONC treated animals, since these waves are supposed to originate from the activity of cells in the INL and GCL^39^. The fact that hypothermic treatment was able to significantly normalize all parameters points to a clear benefit for this therapeutic option.

Gene expression data for CIRP and RBM3 confirmed their involvement in cellular stress and hypothermia, respectively. There was a significant CIRP increase due to trauma but cold exposure did not increase CIRP expression significantly. It has been shown before that CIRP is regulated by cold but also by other cellular stressors such as hypoxia and U.V. light exposure^32, 34, 46^. Our data suggest that CIRP expression responds to cell damage, as well. On the other hand, RBM3 expression was not affected by IONC but responded nicely to hypothermia both in the sham and the IONC-operated eyes, thus confirming its role as a CSP. Previous studies have demonstrated that CSP elevation results in the upregulation of antiapoptotic proteins such as BCL2 and/or a downregulation of proapoptotic proteins such as BAX, BAD, BAK, and others, protecting cells and tissues from programmed cell death^36, 37^. We have shown that the antiapoptotic mediator BCL2 was diminished in IONC-treated eyes, which goes in parallel with the observed decrease in the number of RGCs, the loss of retinal electrical activity, and visual loss. Interestingly, hypothermia completely prevented BCL2 downregulation in the posterior chamber of traumatic eyes whereas expression of the proapoptotic protein BAD did not change. These data suggest a potential molecular mechanism underlying the beneficial effects of therapeutic hypothermia in the eye: i) cold exposure induces the expression of CSPs, ii) these more abundant CSPs bind the mRNA of antiapoptotic proteins, iii) this binding regulates the half-life of the mRNAs and thus their bioavailability for protein synthesis, and iv) as a result, the apoptotic process gets inhibited and visual neurons survive the retrograde insult.

Obviously, additional mechanisms may be playing a role in the beneficial effects of hypothermia in the retina. For example, clinical studies have shown that therapeutic hypothermia cause benefits by down-regulation of oxygen and glucose consumption^47^ or via inhibition of calcium intake by the cells^48^. These aspects should be examined in future IONC studies.

In conclusion, hypothermia constitutes an affordable and efficacious treatment for ocular trauma. The molecular mechanism responsible for this benefit seems to include the upregulation of CSPs and the modulation of the apoptotic cascade, that may lead to the survival of key neuronal populations, such as the RGCs and cells of the INL.

## Methods

### Traumatic injury model and hypothermic treatment

Male young (8 week old) Sprague-Dawley albino rats (n = 94) with genetic quality and sanitary certification from the animal facility of our Institution were cared for in accordance with the guidelines published in the ARVO Statement for the Use of Animals in Ophthalmic and Vision Research. The treatments described below were approved by the Ethical Committee of CICUAL: “Comité Institucional para el Uso y Cuidado de Animales de Laboratorio” (Resolution Nº 255/2014), Facultad de Medicina, Universidad de Buenos Aires, Argentina and by the US Department of Defense (ACURO, protocol number MR130239). Animals were kept under standard laboratory conditions, with light/dark cycles of 12/12 h, and food and water were given *ad libitum*.

The IONC protocol was performed as described^43^, with slight modifications. Briefly, rats were anesthetized with 2% Isofluorane (Baxter, Deerfield, IL) using an E-Z anesthesia vaporizer system (Euthanex, Corp, Palmer, PA). A small incision was performed in the superior temporal quadrant of the left eye to give access to the optic nerve. The optic nerve was crushed with forceps for 60 sec at 1.5 mm from the ocular globe, and the incision was closed with suture. The right eye was sham-operated: the optic nerve was exposed but not crushed. After a period of 45 min of recovery at room temperature (24 °C), some of the rats were subjected to hypothermia in a cold room (8 °C for 3 h) while the rest remained at 24 °C (normothermia). In previous studies we have shown that 3h at 8 °C produces a temperature drop in the rat of approximately 3 °C^35^. To calculate changes in retro-ocular temperature, some animals kept at normothermia (n = 5) and some animals that had just received the hypothermic treatment (n = 5) were deeply anesthetized and a thermocouple (type K, model TPK-01, Tecpel, Taiwan), connected to a digital thermometer (TES-1300, Tecpel), was placed in the orbital fossa and the temperature was rapidly recorded. After surgery, an ointment containing propylene glycol (Systane, Alcon, Buenos Aires, Argentina) was applied on the cornea to prevent desiccation. Rats were given subcutaneous analgesia (Tramadol, Finadiet, Caba, Argentina) the day of surgery and for the next 3 days.

Animals were randomly assigned to 4 experimental groups: i) Control animals (CTL, n = 10) whose both eyes were sham-operated, ii) Control animals subjected to hypothermia (CTL-HYP, n = 10), iii) IONC-treated animals (IONC, n = 10) in which the right eye was sham-operated and the left eye was subjected to IONC, and iv) IONC-treated animals exposed to hypothermia (IONC-HYP, n = 10). For the behavioral tests, some rats (n = 10) were IONC-operated in both eyes. In addition, for TUNEL analysis, some animals of the 4 experimental groups (n = 5 animals per group) were sacrificed at 1, 2, and 6 days after surgery, and for molecular studies some more animals (n = 6 animals per group) were sacrificed just 3 h after temperature treatment.

### Electroretinograms

Thirty days after surgery, rats (n = 10 per experimental group) were subjected to electroretinography, as described^49, 50^. Briefly, after overnight adaptation in the dark, rats were anesthetized under dim red illumination. An ophthalmic solution of 5% phenylephrine hydrochloride and 0.5% tropicamide (Fotorretin, Poen, Buenos Aires, Argentina) was used to dilate the pupils. Rats were placed facing the stimulus at a distance of 25 cm in a highly reflective environment. Scotopic electroretinograms (ERG) were recorded from both eyes simultaneously and 20 responses were collected to flashes of unattenuated white light (1 ms, 1 Hz) from a photic stimulator set at maximum brightness. The registered response was amplified (9 cd s/m^2^ without filter), filtered (1.5-Hz low-pass filter, 500Hz high-pass filter, notch activated), and averaged (Akonic BIO-PC, Buenos Aires, Argentina). The a-wave was measured as the difference in amplitude between the recording at onset and the trough of the negative deflection and the b-wave amplitude was measured from the trough of the a-wave to the peak of the b-wave. To calculate oscillatory potentials (OP), the same photic stimulator was used with filters of high (300 Hz) and low (100 Hz) frequency. The amplitudes of the OPs were estimated by using the peak-to-trough method.

### Behavioral test for visual function

Visual function was assessed using a descending method of limits in a two-choice visual task apparatus designed to determine changes in the dark-adapted luminance sensitivity threshold, as described^51, 52^. Testing in this apparatus occurred in two phases: (i) task acquisition or training, and (ii) visual function testing. The task consisted in finding an escape platform (radius = 6 cm) submerged 2 cm in a circular tank (radius = 60 cm) containing water at 17–22 °C in the shortest possible period of time. Before each session, subjects underwent dark adaptation for 20 min. The two possible platform positions were separated by a dark barrier dividing the tank into a left and right compartment. For visual threshold determinations, the platform illumination was progressively reduced between 10^1.6^ lm/m^2^ and 10^−0.7^ lm/m^2^. The measured behavioral variable consisted of escape latencies, as in previous water maze and visual determination studies in rats^53^.

### Histological analysis and TUNEL

At the end of the behavioral testing, rats were deeply anaesthetized (300 mg/Kg ketamine, Imalgene, Merial Laboratorios, Barcelona, Spain, +30 mg/Kg xylazine, Xilagesic, Proyma Ganadera, Ciudad Real, Spain), and intracardially perfused with 4% paraformaldehyde in PBS. The eyes and optical nerves were postfixed in the same fixative for 24 h at 4 °C, paraffin embedded, and sectioned (3 µm thick). Sections were stained with hematoxylin and eosine. The number of RGCs per 25x objective microscopic field was estimated in each experimental group, as previously reported^54^. Additional retinas, taken from animals sacrificed 6 days after surgery, were stained for terminal deoxynucleotidyl transferase dUTP nick end labeling (TUNEL) with the *In Situ* Cell Death Detection Kit, POD (Roche, Basel, Switzerland), following manufacturer´s instructions.

### RNA extraction and quantitative real-time PCR

A subset of rats (n = 6 per experimental group) were sacrificed 3 h after hypothermic or normothermic treatment. The posterior chambers of the eyes were homogenized with TRIzol (Invitrogen, Madrid, Spain) and RNA was isolated and reverse transcribed as previously reported^35^. Resulting cDNA was mixed with SYBR Green PCR Master Mix (Applied Biosystems, Carlsbad, CA) for quantitative real time polymerase chain reaction (qRT-PCR) using 0.3 µM forward and reverse oligonucleotide primers (Table 1). Quantitative measures were performed using a 7300 Real Time PCR System (Applied Biosystems). Cycling conditions were an initial denaturation at 95 °C for 10 min, followed by 40 cycles of 95 °C for 15 seconds and 60 °C for 1 minute. At the end, a dissociation curve was implemented from 60 to 95 °C to validate amplicon specificity. Gene expression was calculated using absolute quantification by interpolation into a standard curve. All values were divided by the expression of the house keeping gene 18S.

**Table 1:** Primers used for qRT-PCR.

Target	Sequence	Expected band size
CIRP-F	GCATCAGATGAAGGCAAGGT	64 bp
CIRP-R	CCAGCGCCTGCTCATTG	
RBM3-F	TGGAGAGTCCCTGGATGGG	65 bp
RBM3-R	TGGTTCCCCTGGCAGACTT	
BCL2-F	CCGGGAGAACAGGGTATGATAA	81 bp
BCL2-R	CCCACTCGTAGCCCCTCTG	
BAD-F	GCCCTAGGCTTGAGGAAGTC	109 bp
BAD-R	CAAACTCTGGGATCTGGAACA	
18S-F	ATGCTCTTAGCTGAGTGTCCCG	101 bp
18S-R	ATTCCTAGCTGCGGTATCCAGG	

Annealing temperature for all primers was 60 °C.

### Statistical analysis

All data were analyzed with GraphPad Prism 5 software and were considered statistically significant when p < 0.05. Values are expressed as means ± SD. Normally distributed data were evaluated by ANOVA followed by the Dunnet’s (Bonferroni) post-hoc test while data not following a normal distribution were analyzed with the Kruskal-Wallis test followed by the Mann-Whitney U test.
